# Cell-Free Therapies for Chronic Pain: The Rise of the Mesenchymal Stem Cell Secretome

**DOI:** 10.3390/brainsci15121263

**Published:** 2025-11-25

**Authors:** Giada Amodeo, Giulia Galimberti, Stefania Niada, Chiara Giannasi, Elena Della Morte, Silvia Franchi, Benedetta Riboldi, Stefania Ceruti, Anna Teresa Brini, Paola Sacerdote

**Affiliations:** 1 Pain Therapy and Neuroimmunology Unit, Department of Pharmacological and Biomolecular Sciences “Rodolfo Paoletti”, Università degli Studi di Milano, Via Vanvitelli 32, 20129 Milan, Italy; giulia.galimberti@unimi.it (G.G.); silvia.franchi@unimi.it (S.F.); benedetta.riboldi@unimi.it (B.R.); stefania.ceruti@unimi.it (S.C.); paola.sacerdote@unimi.it (P.S.); 2 IRCCS Istituto Ortopedico Galeazzi, 20157 Milan, Italy; stefania.niada@grupposandonato.it (S.N.); chiara.giannasi@unimi.it (C.G.); elena.dellamorte@grupposandonato.it (E.D.M.); anna.brini@unimi.it (A.T.B.); 3 Department of Biomedical, Surgical and Dental Sciences, University of Milan, 20100 Milan, Italy

**Keywords:** conditioned medium, chronic pain, rodent model, in vitro studies, clinical trials

## Abstract

Chronic pain is a pervasive global health issue that significantly impairs quality of life and remains inadequately managed by current therapeutic options. Traditional pharmacological treatments often offer limited relief and are associated with significant side effects, highlighting the urgent need for safer and more effective alternatives. Among emerging strategies, mesenchymal stem cell (MSC)-derived secretome, an acellular product composed of bioactive molecules such as cytokines, growth factors and extracellular vesicles, has gained increasing attention for its potent anti-inflammatory, neuroprotective and immunomodulatory properties. Unlike whole-cell therapies, secretome-based interventions offer advantages, including lower immunogenicity, higher safety and easier standardization and storage. Preclinical studies demonstrated that MSC secretome effectively alleviates pain-like behavior across various models of neuropathic, inflammatory and degenerative pain, primarily through neuroimmune modulation and glial cell reprogramming. In vitro experiments confirm its role in promoting neuronal survival, regulating opioid receptor expression and modulating (neuro)inflammatory responses. Preliminary clinical evidence supports its analgesic efficacy in conditions such as osteoarthritis, chronic low back pain and post-surgical pain, with a favorable safety profile and promising therapeutic outcomes. However, challenges remain, including variabilities in secretome composition, lack of standardized production protocols and absence of large-scale clinical trials. Despite these limitations, MSC secretome therapy represents a transformative approach in pain medicine. Continued research efforts are essential to optimize formulation, dosing and delivery strategies, as well as to clarify the regulatory landscape. With further validation, the MSC secretome could emerge as a novel, scalable and clinically viable solution for the management of chronic pain, bridging critical gaps in current treatment paradigms.

## 1. Introduction

Chronic pain is a significant public health concern, associated with substantial functional impairment and a marked reduction in the quality of life [1,2]. It encompasses a broad spectrum of etiologies, including mechanical injuries, inflammatory processes, metabolic disorders (e.g., diabetes), infections and cancer. When nerve damage is involved, due to chemotherapy, viral infections or trauma, it is classified as neuropathic pain [3,4,5]. Irrespective of its origin, chronic pain arises from a complex interplay of biological, psychological and social factors. Mechanistically, it involves peripheral and central sensitization, wherein nociceptors and central neural circuits exhibit hyperexcitability and undergo maladaptive plastic changes, including synaptic reorganization in the spinal cord and brain [6,7]. Neuroimmune interactions further contribute to pain persistence: activated glial cells release pro-inflammatory cytokines that amplify nociceptive transmission and promote neural remodeling [8]. Additionally, chronic psychosocial cues, such as trauma, mood disorders or prolonged stress, can lead to neuroendocrine and immune dysregulation, fueling neuroinflammation and central sensitization [9].

**Figure 1:**
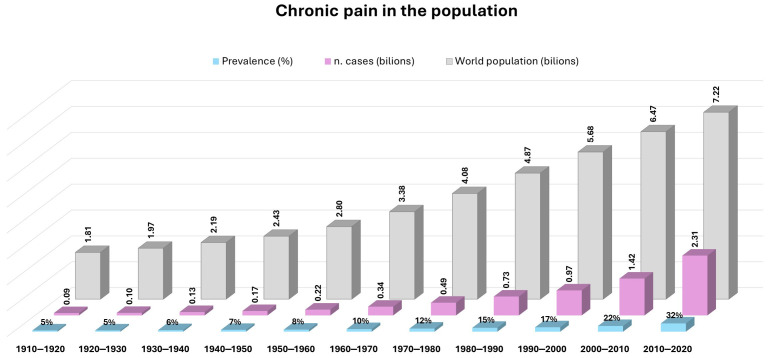
Chronic pain in the global population. The graph was created by the authors using Excel, based on data from [16,17,18,19,20].

The global burden of chronic pain is enormous, affecting nearly 30% of the population worldwide. The percentage of patients with pain continues to increase, due both to demographic expansion and to the progressive aging of the population, with the number of diagnosed cases currently approaching 2.5 billion (Figure 1) [10,11,12,13]. Consequently, chronic pain is now recognized as a public health epidemic. Beyond causing physical disability, it also profoundly impairs social functioning, with more than 60% of affected individuals experiencing comorbid anxiety, depression or stress-related symptoms [1,14,15].

Several drug treatments are available and must be carefully selected based on the type and pathophysiology of pain, as well as the individual characteristics of the patient. Current treatment approaches for chronic pain include pharmacotherapy, e.g., non-steroidal anti-inflammatory drugs (NSAIDs), opioids, antidepressants (tricyclics and serotonin-norepinephrine reuptake inhibitors (SNRIs)), anticonvulsants (gabapentin and pregabalin) and topical agents, as well as non-pharmacological methods such as transcutaneous electrical nerve stimulation, spinal cord stimulation and cognitive behavioral therapy [21]. Nevertheless, efficacy remains modest; only a minority of patients achieve meaningful relief, while adverse effects are frequent and sometimes severe, limiting the duration of therapy [12,22,23,24,25]. Evidence indicates that many commonly used medications yield only moderate benefit, with effect sizes fading over time, and intolerable side effects causing up to 14% of patients to discontinue therapy or rate treatments as inadequate [2,12,23]. Despite the ever-deepening understanding of the mechanisms underlying chronic pain, translational progress is still limited [3,24,26]. Consequently, management of chronic pain remains a global challenge, demanding novel strategies to improve patient outcomes and quality of life. These enduring treatment gaps highlight an urgent need for safer, more effective and personalized interventions.

## 2. Novel Therapeutic Strategy

Considering the limited efficacy and significant adverse effects associated with conventional pharmacological treatments, growing attention is being directed toward novel therapeutic approaches for the management of chronic pain. These emerging strategies aim to target specific pathophysiological mechanisms involved in pain chronicization and neural sensitization, offering the potential for more precise, effective and better-tolerated interventions. Advances in neurobiology and molecular medicine have led to the development of agents targeting ion channels (like voltage-gated sodium channel (Nav1.7 or Nav1.8) antagonists and transient receptor potential vanilloid (TRPV) 1 modulators), neuroinflammation modifiers (including glial inhibitors and cytokine blockers) and monoclonal antibodies against nerve growth factors (anti-NGF antibodies) [27,28]. Furthermore, gene therapy and RNA-based treatments are being explored for their ability to modulate pain-related gene expression in a targeted and long-lasting manner [29]. Non-invasive neuromodulation techniques, such as transcranial magnetic stimulation (TMS) and transcranial direct current stimulation (tDCS), are also gaining traction as potential tools for altering central pain processing circuits without systemic side effects [30]. In parallel, advances in digital health facilitate the integration of digital therapeutics, wearable devices and artificial intelligence-driven platforms to enable personalized pain monitoring and behaviorally based interventions [31]. These innovations, along with precision medicine approaches based on phenotyping and biomarkers, mark a paradigm shift in chronic pain management and represent a hopeful direction for improving therapeutic outcomes in affected individuals. In addition to these pharmacological, genetic and technological innovations, increasing emphasis has been placed on biological strategies that harness the regenerative and immunomodulatory properties of stem cells and their secretome, also known as conditioned medium (CM). These cell-free approaches represent a promising frontier in chronic pain management, as they offer the potential to modulate neuroinflammation, neuronal survival and sensitization processes while overcoming many of the limitations associated with conventional cell-based therapies [32].

## 3. Secretome

**Figure 2:**
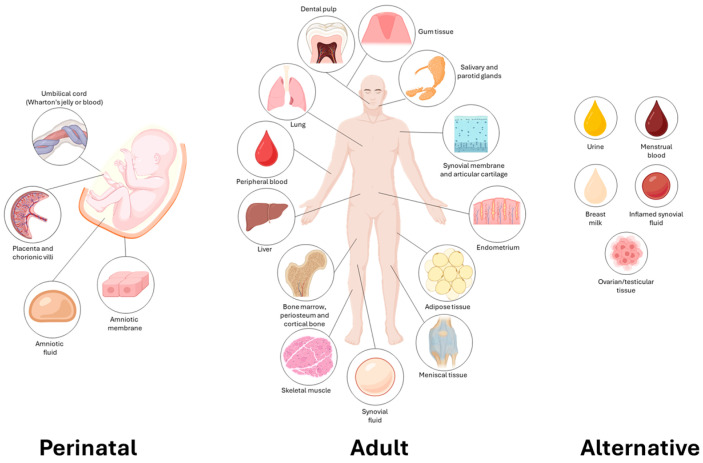
Overview of the mesenchymal stem/stromal cells (MSCs) sources. Perinatal MSCs are derived from birth-associated tissues [59,60,61,62,63,64] and show high proliferation and strong immunomodulatory activities. Adult MSCs come from postnatal tissues [65,66,67,68,69,70,71,72,73,74,75,76,77,78,79] and mainly support maintenance and repair with lower activities on proliferation, while alternative MSCs originate from unconventional sources [48,80,81,82,83] and offer accessible and promising regenerative potential. These different origins confer distinct biological and functional properties that may be exploited for regenerative and therapeutic applications. This image was drawn by the authors using BioRender (https://www.biorender.com/, accessed on 15 October 2025) (licensed to the University of Milan) and further edited in post-production using PowerPoint and a graphic tablet.

Secretome is an acellular biological product containing soluble factors, such as growth factors, cytokines and chemokines, microRNAs (miRNAs) and lipids, as well as extracellular vesicles (EVs), including exosomes, microvesicles and apoptotic bodies secreted by stem cells [33,34]. All these factors, which mediate most of the paracrine effects traditionally attributed to stem cell therapy, collectively influence pain-related signaling cascades, glial cell activation and peripheral and central sensitization mechanisms [33,35]. Unlike whole-cell therapies, secretome-based approaches offer several advantages, including reduced immunogenicity, easier storage and standardization, no risk of tumor formation and the ability to fine-tune their composition for targeted effects [36,37,38,39]. Among the various secretomes investigated, mesenchymal stem cell (MSC)-derived secretome remains the most extensively studied and widely applied, owing to the ease of MSC isolation from multiple tissue sources and the unique tissue-specific characteristics that each MSC population exhibits (Figure 2; Table 1). These properties not only enhance the versatility of MSC secretome but also allow for tailoring secretome-based therapies according to the pathological context and therapeutic goal [34,40,41,42,43,44,45,46,47,48]. However, in addition to the MSC secretome, on which this review will focus, also secretome derived from other stem cell types, including neural stem cells (NSCs), induced pluripotent stem cells (iPSCs) and embryonic stem cells (ESCs), has demonstrated bioactive profiles capable of modulating neuroinflammation, promoting neuronal survival and facilitating synaptic plasticity [33,36,49]. For example, NSC-derived secretomes have shown promising results in reducing neuropathic pain behaviors in animal models by suppressing spinal cord microglial activation and pro-inflammatory gene expression [50]. Similarly, iPSC-derived exosomes are being explored for their regenerative and immunomodulatory potential, with initial data suggesting a favorable safety profile and the capacity to deliver neuroprotective cargo across the blood-brain barrier [51]. However, these secretomes may contain factors associated with uncontrolled proliferation or inappropriate differentiation, raising safety concerns for clinical translation [52]. These concerns are particularly relevant for pluripotent stem cell–derived secretomes, such as those obtained from iPSCs or ESCs, which, due to their pluripotent nature, generate a highly enriched but less predictable secretome. This milieu includes potent growth factors such as fibroblast growth factor (FGF)2, VEGF (vascular endothelial growth factor) and IGF (insulin-like growth factor), as well as signaling modulators like Wnt and Hedgehog proteins, together with miRNAs that sustain pluripotency or induce dedifferentiation. Collectively, these components exert strong pro-proliferative and pro-differentiative activities, thereby raising the potential risk of aberrant proliferation or even indirect tumorigenesis [53,54]. Similarly, NSC secretomes are enriched in neurotrophic factors such as BDNF (brain-derived neurotrophic factor) and NGF, which support neurogenesis, synaptogenesis and neuronal survival, but also contain signaling molecules that can stimulate glial and neuronal proliferation. While these properties make NSC-derived conditioned media promising candidates for neuroprotection, if not tightly regulated, their bioactive cargo may drive glial hyperplasia, maladaptive circuit remodeling and glial scarring, ultimately resulting in dysregulated tissue growth [55,56]. In contrast, MSC-derived secretomes predominantly reflect immunomodulatory and reparative functions, being enriched in anti-inflammatory cytokines (e.g., transforming growth factor-beta (TGF-β) and interleukin (IL-) 10), anti-apoptotic and pro-regenerative factors. Compared with pluripotent or neural stem cell secretomes, MSC secretomes display lower variability, a more favorable immunological profile and an absence of high levels of strongly mitogenic signals, features that confer greater stability and a superior safety profile for clinical translation [57,58].

### Mesenchymal Stem Cell (MSC)-Derived Secretome

The MSC secretome is increasingly recognized as one of the most advantageous platforms for cell-free therapies, owing to its reproducible composition, broad spectrum of bioactivities, safety, accessibility and immunomodulatory profile of its cellular sources [33,84,85,86,87,88,89]. Indeed, MSCs can be readily isolated from adult tissues (such as bone marrow, adipose tissue or umbilical cord) with minimal ethical concerns and a lower risk [87,90]. The MSC secretomes comprise a complex mixture of soluble molecules, anti-inflammatory cytokines (e.g., IL-10 and TGF-β), neurotrophic factors (e.g., BDNF, NGF and GDNF (glial cell line-derived neurotrophic factor)) and EV vesicles enriched in regulatory miRNAs. Collectively, these components exert potent immunomodulatory, anti-apoptotic, pro-regenerative, neuroprotective and paracrine effects [85,86,87,88,90,91,92,93]. Additionally, MSCs possess intrinsic immune privilege, enabling the use of their secretome in allogeneic contexts with minimal risk of immune rejection, a unique property that allows secretome from different donors to be used without requiring autologous stem cells [89]. Interestingly, the composition of the MSC secretome can be modulated in vitro through a process called preconditioning/priming/licensing. For example, we demonstrated that a brief 5 min cytokine stimulus can “activate” adipose-derived MSCs (ASCs), significantly altering their secretome, which becomes enriched in extracellular particles and diverse immunomodulatory factors [94]. Similarly, other studies have shown that cytokine-primed/licensed MSCs change their secretory profile also in terms of miRNA content and acquire distinct immunomodulatory properties on macrophage polarization and peripheral blood mononuclear cells (PBMCs) regulation [93,95,96]. Furthermore, González Rodríguez and colleagues [97] compared different priming strategies—including hypoxia, inflammatory stimuli and 3D culture conditions—demonstrating that both individual and combined stimuli modulate secretome composition, with hypoxia-primed cells displaying superior immunomodulatory activity in functional in vitro assays.

Overall, these properties make the MSC secretome a particularly attractive, scalable and clinically viable candidate for cell-free pain therapies [98]. Indeed, although still in preclinical or early translational stages, secretome-based therapies represent a promising and versatile platform for pain modulation, paving the way for next-generation biological drugs capable of overcoming the limitations of conventional pharmacological treatments.

Therefore, this narrative review aims to provide a comprehensive overview of in vitro, preclinical and clinical studies examining the effects of MSC-derived secretome on chronic pain, highlighting its therapeutic potential in alleviating pain and modulating nociception and neuroinflammation.

## 4. Methodology

This work was conducted as a narrative review aimed at providing an overview of preclinical and early clinical evidence on MSC secretome in chronic pain. A search was conducted in July 2025, according to the following criteria:

(a) Filter time: none.
(b) Database: PubMed, Cochrane Library and Embase.
(c) Language: English.
(d) Keywords: terms used were “chronic pain” AND “mesenchymal cells” AND “secretome”, “conditioned medium”, “acellular therapy”, “extracellular vesicles” or “exosomes”.
(e) Key terms were searched in the title, keywords and abstract. The search was open to all parameters in order to avoid information loss. MeSH (Medical Subject Headings) terms were not used.
(f) The papers’ selection was based on critical reading.

The first co-authors independently reviewed the titles and abstracts of all retrieved articles. Abstracts of the selected papers were then examined to determine whether they met the inclusion criteria. Studies were included if the effect of MSC-derived acellular strategies on pain represented the primary, secondary or exploratory outcomes. Conversely, studies investigating MSC-derived acellular strategies exclusively for tissue regeneration, repair or inflammation without pain evaluation were excluded. In addition to the electronic search, a manual screening of the reference lists and related publications of the selected articles was performed to ensure that no relevant studies were overlooked. Overall, a total of 147 manuscripts were included, encompassing both the original studies specifically addressing the focus of this narrative review (n = 28) and additional papers used to provide background and contextual information.

## 5. Dissecting the Evidence: From Petri Dish to Complex Organisms

As the therapeutic potential of the MSC secretome continues to gain traction, an examination of current experimental evidence becomes imperative [99,100,101]. The following sections delve deeper into the experimental landscape that underpins this emerging therapeutic approach. The different sections examine: (i) studies in in vivo preclinical models, where the analgesic efficacy of secretome-based interventions has been consistently validated in neuropathic or other chronic pain conditions; (ii) in vitro studies, which have provided critical insights into the cellular and molecular interactions between the secretome and target immune or neural cells; (iii) the first clinical trials about the secretome efficacy for pain management. Together, these subsections form a cohesive and timely framework for understanding how secretome-based therapies may reshape the paradigm of pain treatment.

### 5.1. In Vivo Preclinical Studies

The therapeutic use of MSCs-derived secretome has garnered increasing attention due to its analgesic and anti-neuroinflammatory properties in several rodent models of chronic pain. The key findings of these studies are summarized in Table 2.

The paracrine action of MSCs had been hypothesized for years, but it was not until 2017 that the synergistic collaboration between our research team, led by Prof. Sacerdote and Prof. Brini’s group, provided the first experimental evidence that the secretome alone could exert robust and long-lasting analgesic effects in vivo through neuroimmune modulation [102]. In this study, they demonstrated that not only MSCs, but also their secretome was able to rapidly, durably and significantly reverse mechanical allodynia and thermal hyperalgesia in a streptozotocin (STZ)-induced diabetic neuropathy mouse model. Interestingly, a single administration of secretome, given at either early or advanced stages of the disease, produced a sustained reduction of sensory hypersensibility, with significant pain relief lasting for over two months compared to pathological controls. Moreover, analgesic benefit was associated with the downregulation of pro-inflammatory cytokines (such as IL-1β, IL-6 and tumor necrosis factor (TNF)-α) and the upregulation of anti-inflammatory IL-10 within the peripheral and central nervous system. The study established a clear link between secretome-based therapy and neuroimmune modulation. Afterwards, other research groups corroborated the analgesic efficacy of MSC secretome in diabetic neuropathy models. Indeed, another study showed that cell-free therapy similarly reverted pain-like behaviors in the STZ model [104]. Moreover, in a spontaneous diabetic neuropathy model (the db/db mouse), the early-stage treatment with MSC secretome improved both thermal and mechanical sensitivity, restored intraepidermal nerve fiber density, reduced neuronal and Schwann cell apoptosis, enhanced angiogenesis and alleviated neuroinflammation in peripheral nerves [108]. Fan and colleagues further advanced this finding, confirming the therapeutic potential of MSC EVs in db/db mice. EVs injection was able to modulate both pain and neuroinflammation, reinforcing the central role of paracrine signaling. Moreover, this research team demonstrated that engineering exosomes (enriched in miRNA-146a) significantly enhanced their analgesic and anti-neuroinflammatory efficacy [109,111].

Subsequently, different research groups evaluated the analgesic and anti-neuroinflammatory effect of MSC secretome treatment, also in several traumatic neuropathic pain models, demonstrating that both whole secretome and EV-based treatments, administered either intravenously, intraperitoneally or intrathecally, significantly alleviated mechanical allodynia, shifted the cytokine profile toward an anti-inflammatory state, modulated oxidative stress and neurotrophic signaling, promoted microglial polarization toward the anti-inflammatory phenotype and restored autophagic pathways, overall reducing neuroinflammation [105,107,112,113,114,115,118,119]. Additionally, a secretome enriched with anti-inflammatory cytokines and miRNAs demonstrated enhanced analgesic efficacy when administered as a preventive treatment [120].

Beyond neuropathic pain models, the application of MSC secretome has also been explored in osteoarthritis (OA). However, the majority of research in OA models has focused on the secretome regenerative and anti-inflammatory actions within the joint environment, whereas only a few studies have directly assessed its effects on pain, despite the latter being a highly clinically relevant feature of OA. Among these, Khatab and colleagues were the first to demonstrate that local (intra-articular) injection of MSC secretome in OA knee effectively reduced pain. However, the treatment did not result in any detectable improvement in joint structural integrity [106]. In parallel, our study demonstrated that the route of MSC secretome administration is important for relieving pain and exerting anti-neuroinflammatory effects in a knee OA model. Specifically, although intravenous, intraplantar and intra-articular administration attenuated thermal hyperalgesia and mechanical allodynia and reduced the expression of neuroinflammatory mediators in both the peripheral and central nervous system, the intravenous route proved to be the most effective, while the intra-articular one was the least [110]. Subsequently, another study provided evidence that MSC secretome administration mitigated pain, inflammation and counteracted structural degeneration in a temporomandibular joint OA model [117].

Finally, the therapeutic effect of MSC EVs was also demonstrated in a model of interstitial cystitis bladder pain syndrome, where their administration suppressed mechanical allodynia and glia activation by inhibiting NLRP-3 (NOD-like receptor family pyrin domain-containing)inflammasome activation, a critical driver of neuroinflammation [116]. Additionally, it should be noted that we identified a single study reporting that, in contrast to MSC treatment, the secretome failed to induce any improvement in pain-like behavior in a rat model of chemotherapy-induced peripheral neuropathy (CIPN) [103]. Based on our experience, this lack of efficacy could be partially attributed to the secretome processing method used. Indeed, the secretome was lyophilized prior to administration. Similarly, our group also found that lyophilization of the secretome followed by rehydration produced only modest and short-lived analgesic effects (unpublished data). It could therefore be hypothesized that degradation or loss of key bioactive components occurs during the drying process.

The variability across studies indicates that the analgesic efficacy of MSC secretome could be strongly influenced by technical and experimental parameters, including the cellular source, the methods of secretome processing and storage (e.g., freeze-dried versus frozen), the route of administration, the timing of delivery and the type of formulation used (e.g., whole secretome versus EVs). However, although these studies differ significantly in terms of pain models used, MSC sources, routes of administration, dosages and whether whole secretome or fractions (EVs) were applied, all their findings clearly demonstrated the neuroimmune-modulating properties of secretome in a wide range of pain conditions and also elucidate the key common molecular mechanisms, including: i. suppression of pro-inflammatory cytokines and enhancement of anti-inflammatory mediators (e.g., IL-10 and TGF-β); ii. inhibition of microglial and astrocytic activation, with a phenotypic shift toward a homeostatic state; iii. downregulation of pain-sustaining signaling pathways (e.g., purinergic P2X4/P2X7 receptors, Toll-like Receptors (TLRs) and PI3K/AKT/mTOR); iv. upregulation of neurotrophic factors such as BDNF and VEGF in the peripheral and central nervous systems.

Thus, although further studies in this field still need to be done, secretome-based therapy represents a promising acellular alternative in chronic pain treatment, offering immunomodulation, neuroprotection and regenerative support without any risks associated with cell transplantation. Finally, it is worth noting that none of these studies examined the psychological dimension of pain. This aspect has never been specifically investigated, although some preclinical studies have explored the effects of MSC secretome on mood and psychiatric disorders [121,122,123,124,125,126,127,128,129], suggesting that secretome treatment could not only alleviate pain but also reduce the associated emotional burden.

### 5.2. In Vitro Studies

To better understand the molecular basis underlying the analgesic and neuroprotective effects of MSC secretome observed in vivo, several in vitro studies have been performed over the past years. These investigations provide a controlled environment to dissect the direct interactions between secretome components and target cells, such as neurons, glial cells or immune cells, which are often dysregulated in chronic pain states. Such models allow for high-resolution analysis of cellular responses, spanning from cytokine expression and signaling pathway modulation to cell survival, neurite outgrowth and phenotypic changes in microglia or astrocytes. In particular, in vitro experiments could prove essential for identifying the specific molecular mediators responsible for the immunomodulatory and anti-inflammatory actions of secretome-based therapies, as well as for optimizing formulation parameters and evaluating dose–response relationships. The studies described below, and summarized in Table 3, provide important mechanistic insights into how secretome-based interventions may counteract the cellular processes that drive chronic pain.

The research group led by Dr. Ezquer has focused extensively on investigating the therapeutic potential of the MSC secretome in diabetic polyneuropathy. In their initial work, they demonstrated that in vitro preincubation of dorsal root ganglia (DRG) neurons with MSC secretome effectively counteracted apoptosis induced by high-glucose exposure, providing evidence of its neuroprotective capacity [130]. Building on these findings, the group conducted more in-depth ex vivo analyses. First, they examined DRG neurons harvested from db/db mice that had previously received in vivo treatment with MSC secretome. When cultured in vitro, these neurons exhibited significantly enhanced neurite extension and arborization, indicating that prior in vivo exposure to secretome positively influenced axonal regeneration [108]. In a subsequent study, they assessed the in vitro effects of secretome on DRG neurons isolated from db/db mice, observing a modest yet significant increase in neurite outgrowth and branching compared to untreated diabetic neurons [131]. Moreover, in their studies, the authors employed various preconditioning strategies to stimulate MSCs to secrete bioactive factors, aiming to enhance the therapeutic potential of their secretome. In their results, authors also reported that deferoxamine, an iron chelator, induces a hypoxia-like stimulus characterized by controlled and moderate cellular stress. This condition enhances the release of trophic and anti-inflammatory factors, thereby potentiating secretome activity both in vitro and in vivo [130].

Furthermore, chronic pain conditions may be associated with a reduction in the availability and expression of opioid receptors, thereby affecting analgesic responsiveness to opioids such as morphine. In particular, PET (positron emission tomography) analysis in patients suffering from chronic pain documented a decreased availability of Mu opioid receptors (MORs) compared to healthy controls, suggesting a reduction in receptor expression or trafficking within brain regions involved in pain processing [135]. Additionally, inflammatory and hypoxic conditions commonly associated with chronic pain can alter the expression of all major opioid receptor subtypes (MORs, Delta (DORs) and Kappa (KORs)) in both the peripheral and central nervous systems, thereby modulating neuronal sensitivity to pain and analgesia [136]. Notably, in nociceptors, persistent stimulation can trigger the translocation of DORs from intracellular compartments to the plasma membrane, enhancing sensitivity to DOR agonists and potentially contributing to opioid tolerance [137]. These findings help to explain the reduced efficacy of both endogenous and pharmacological analgesia observed under pathological pain conditions. In this context, a recent study investigated the potential of the MSC secretome in modulating opioid receptor expression [132]. Researchers employed cerebral organoids (CeOs), self-organizing, multicellular and 3D structures that recapitulate key features of the human brain. Treatment with MSC secretome resulted in a significant protein upregulation of MORs beyond physiological value, suggesting a possible role in pain signaling modulation. Additionally, secretome treatment promoted neurogenesis and astrogliogenesis without altering dopamine levels, which may indicate a therapeutic benefit in the context of opioid addiction (e.g., as a co-treatment). The study also compared the neuromodulatory effects of the secretome with those of two opioids, morphine and buprenorphine, commonly administered for pain management. These findings highlight the potential of MSC secretome not only in enhancing opioid receptor signaling and analgesic efficacy but also as a promising adjunct therapy to mitigate the side effects and limitations of conventional opioid treatments [132].

In order to investigate in vitro the effects of treatments relevant to chronic low back pain, the efficacy of the cell-free therapy was also evaluated in annulus fibrosus (AF) cells, which constitute the outer portion of the intervertebral disc obtained from biopsies of patients with idiopathic scoliosis or disc degeneration. Cells were exposed to four experimental conditions [133]: (i) no additional stimulation, (ii) mechanical stress, (iii) IL-1β exposure and iv) combined mechanical stress with IL-1β.

The secretome treatment was tested in both the unstressed condition and under dual stress. In the unstressed condition, secretome application led to mixed results regarding inflammation. In fact, it induced a synchronous increase in IL-6, IL-8 and Prostaglandin (PG) E_2_, typically indicative of a pro-inflammatory response, alongside an upregulation of Custer of Differentiation (CD)55, a regulatory mediator known to promote anti-inflammatory phenotypes. However, the simultaneous upregulation of Matrix Metalloproteinase (MMP) 1 and MMP3, together with increased expression of Tissue Inhibitor of Metalloproteinases (TIMP) 1 and TIMP2, suggested an extracellular matrix remodeling activity that may support tissue regeneration. The elevated elastin levels further indicated a pro-regenerative and structurally supportive environment. Instead, under dual stress conditions, secretome treatment resulted only in a reduction of MMP1 levels alongside an increase in TIMP1, suggesting a decreased extracellular matrix degradation activity and a shift toward a more restrained remodeling environment. These data suggested secretome potential to modulate inflammation and extracellular matrix remodeling to support tissue repair while limiting excessive degradation [133]. Subsequently, another research group employed a similar in vitro model, again aiming to simulate chronic low back pain (discogenic pain) [134]. Nucleus pulposus and annulus fibrosus cells derived from healthy donors were exposed to TNF-α–induced stress and then treated with whole secretome, EVs alone or EV-depleted secretome. All three treatments promoted a highly synergistic anti-inflammatory modulation; however, the whole secretome exerted the most pronounced effects. This superior efficacy is likely attributed to the broader range of bioactive components it contains, including soluble proteins, freely dissolved factors, nucleic acids and lipids. Moreover, the whole secretome also emerged as a promising therapeutic strategy for counteracting disc degeneration. These results further support the therapeutic potential of the MSC secretome, particularly in its complete form, as a multifaceted tool for future clinical application in modulating inflammation and preventing disc degeneration in chronic low back pain conditions [134].

Finally, lipopolysaccharide (LPS) stimulation was applied to an immortalized microglial cell line, a widely accepted and validated experimental paradigm for inducing neuroinflammation, a hallmark feature of chronic pain [138,139]. Treatment with EVs was found to reduce the overexpression of pro-inflammatory cytokines and microgliosis. Moreover, in this study, the authors identified Rsad2 (Radical S-Adenosyl Methionine Domain-containing 2), a gene encoding the antiviral protein viperin, which is involved in immune regulation and microglial activation, as a key mediator of the observed effects. Indeed, Rsad2 silencing was able to counteract microglial activity by blocking the TLR2/MyD88/NF-κB signaling pathway, resulting in a significant reduction in pro-inflammatory cytokine production. Thus, the authors demonstrated that EV treatment produced effects similar to those achieved through direct Rsad2 silencing, suggesting that EVs exert their anti-inflammatory effects, at least in part, through the modulation of Rsad2 expression [118].

These in vitro studies collectively underscore the multifaceted therapeutic potential of MSC secretome in modulating inflammation, promoting neuroprotection and counteracting the cellular mechanisms underlying chronic pain.

### 5.3. Clinical Reports

In the last years, the use of MSC-derived secretome for pain management in patients has been documented, and a summary of the available clinical studies is provided below (Table 4) [140,141,142,143]. Of note, although several clinical trials include pain-related outcomes, none have been specifically designed or registered with the primary aim of investigating the analgesic potential of secretome-based therapies. However, when the terms “secretome” or “conditioned medium” were searched under the filter “interventional” as study type, without including the term “pain”, a total of 87 results were identified (47 for secretome and 40 for conditioned medium). After removing duplicates and manually reviewing each trial, 52 unique studies remained, which are listed in Supplementary Table S1. These trials span multiple medical fields, mainly dermatology, neurology and orthopedics, and further support the rationale for considering cell-free products as valid therapeutic alternatives. Among these, four studies include pain as a secondary or exploratory outcome: NCT06688318 and NCT05579665, which evaluate secretome alone and NCT05909488 and NCT04314661, which assess its effects in combination with cell therapy. Their main characteristics are summarized in Table 5. To date, the only completed trial with publicly available results is the study by Partan et al. [141], which will be discussed in detail later.

In a study, 16 patients with musculoskeletal pain in various anatomical regions were enrolled. Local administration of ASC secretome was performed in a total of 27 body sites, with the number of treated sites varying among patients. The secretome was derived from healthy donor tissue, processed in the laboratory and therefore administered in an allogeneic manner. For each patient and each treated site, pain was assessed using a Numeric Rating Scale (NRS), which evaluated two conditions: the “current pain state” and the “worst pain state in one week,” both prior to administration and at 15 min, 1 day, 1 week and 4 weeks post-injection. Some patients received a second secretome administration, for a total of 7 additional painful sites treated. In most anatomical regions, secretome treatment resulted in pain reduction as early as 15 min after injection. The study demonstrated that the severity of both the “current pain” and the “worst pain in one week” significantly decreased at all follow-up timepoints and remained lower than baseline values. Moreover, the second administration further reduced pain levels over the 4-week follow-up period, highlighting the rapid and sustained efficacy of secretome treatment for pain management [140].

An open-label clinical study involving 30 patients with grade 2 or 3 knee OA, as classified by the Kellgren and Lawrence (KL) radiographic scale, was conducted to compare the effects of umbilical cord-derived mesenchymal stem cell (UC-MSC) secretome versus hyaluronic acid (HA). Each participant received intra-articular injections of either 2 mL of UC-MSC secretome or 2 mL of HA once a week for five consecutive weeks. The secretome was produced and packaged at the Regenic PT. Bifarma Adiluhung laboratory, which is certified for Good Manufacturing Practices (GMP) by the Indonesian Food and Drug Authority [141]. Pain levels were assessed using the Visual Analog Scale (VAS), while OA severity was evaluated through the Western Ontario and McMaster Universities Osteoarthritis Index (WOMAC). Investigations were performed at baseline, weekly during the five-week treatment period and at a final follow-up conducted 12 weeks after treatment initiation. Although both treatments led to a progressive and effective reduction in pain, remaining significant at week 12, the secretome treatment demonstrated superior efficacy. Additionally, serum levels of MMP3 and TGF-β were measured before treatment and at the final follow-up. While both groups showed a decrease in the MMP and an increase in the anti-inflammatory cytokine, the changes were significantly greater in the secretome group compared to the HA group. Therefore, UC-MSC secretome may offer a more potent therapeutic alternative to HA in reducing pain and modulating inflammation in OA patients [141].

In a case report, a single patient with chronic low back pain underwent percutaneous laser disc decompression (PLDD) from L2 to L4, followed by the administration of UC-MSC secretome in the vertebrae from T12 to S5, the epidural region of the sacral spine and the piriformis muscles. The study did not provide information on the origin of the secretome used. Pain was assessed using a VAS before and one week after surgery. A significant reduction in pain was observed (VAS score reduced from 5 to 2). Follow-up assessments were also performed at 3 and 6 months, assessing both pain and spinal structure (radiographic imaging), showing notable improvements in postural stability, sustained pain relief and increased bone mineral density in the lumbar spine, suggesting an ongoing spinal regenerative process. Although, due to the combined nature of the intervention, it is not possible to determine the specific contribution of PLDD versus secretome treatment, this multimodal approach demonstrated a favorable safety profile that may represent a promising option for future clinical applications [142].

Finally, a randomized controlled clinical trial was conducted involving 15 patients with total brachial plexus injury. Patients underwent nerve transfer surgery and were treated immediately afterwards at the neuromuscular junction of the median nerve–flexor digitorum superficialis muscle with either UC-MSCs or their secretome. The MSCs and their secretome derived from healthy donor tissue were processed in the laboratory and therefore administered in an allogeneic manner. The study reported significant postoperative improvements in all patients across multiple parameters, including physical functioning, role limitations, energy/fatigue, emotional well-being, social functioning, pain, general health and DASH (Disabilities of the Arm, Shoulder and Hand) scores. No statistically significant differences were observed between the two patient cohorts. This investigation suggests that cell-free therapy may represent a valid alternative to stem cell-based treatment and that, in this context, a combined approach (surgical intervention plus biological treatment) may offer safe clinical benefits [143].

Overall, although limited, these clinical investigations demonstrate that MSC-derived secretome may exert a rapid, sustained and clinically significant analgesic effect in a wide range of pain-related disorders, from OA and chronic low back pain to post-surgical and neuropathic pain. The favorable safety profile, combined with its comparable efficacy to cell-based treatments, supports the growing interest in secretome-based interventions as a valid alternative or adjunct to conventional and regenerative pain management strategies. Importantly, none of the clinical studies cited above reported any adverse effects associated with secretome administration. Nonetheless, further large-scale, standardized clinical trials are warranted to fully elucidate the mechanisms of action, optimal dosing regimens and long-term outcomes associated with secretome therapy.

## 6. Conclusions

MSC-derived secretome has emerged as a promising cell-free therapeutic strategy, offering regenerative, anti-inflammatory and immunomodulatory effects across a wide range of clinical conditions. In the context of pain management, both preclinical and clinical data increasingly support the ability of MSC secretome to modulate pain pathways and promote tissue repair without the risks associated with live cell transplantation. However, it is important to distinguish between mechanistic plausibility and demonstrated clinical benefits. While preclinical studies provide compelling evidence for neuroimmune modulation and analgesic effects through multiple molecular pathways, human data remain preliminary, heterogeneous in product characterization and derived mainly from small, open-label studies.

Compared to conventional MSC-based therapies, secretome administration offers distinct advantages, including lower immunogenicity, potentially standardized production, easier storage and transport and higher safety in terms of tumorigenicity and vascular occlusion. These benefits, combined with growing evidence of efficacy, position secretome-based therapies as attractive candidates for the treatment of pain of several origins.

**Figure 3:**
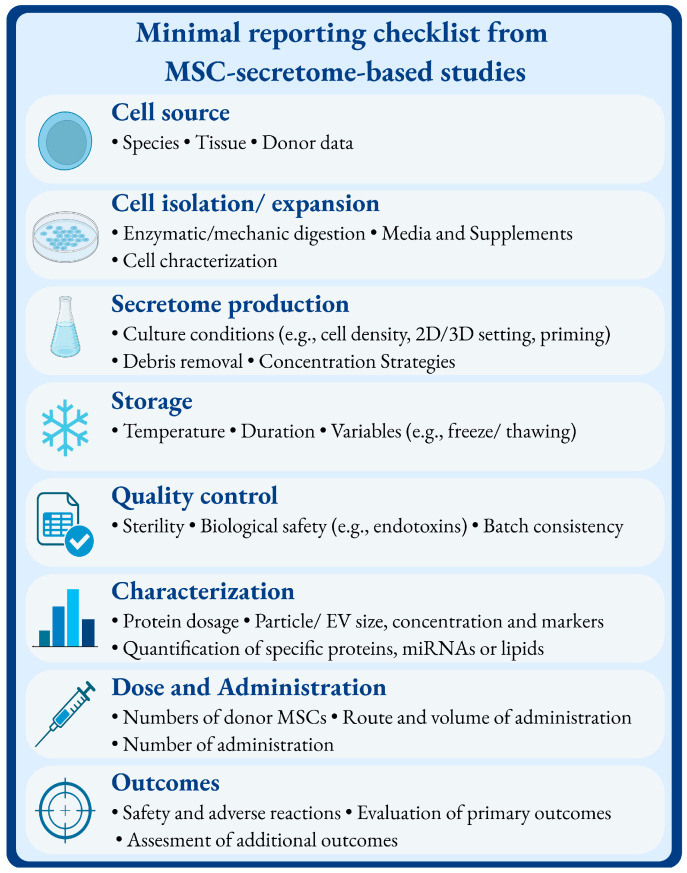
Summary of key methodological parameters in MSC secretome-based studies. This figure summarizes the essential methodological aspects that should be reported in studies investigating MSC-derived secretome-based approaches. It includes parameters related to the cell source, isolation and expansion, secretome production, storage, quality control, characterization, dose and administration, and outcome assessment. Together, these elements contribute to ensuring reproducibility, transparency and comparability across studies in the field of MSC-derived acellular therapies. Created in BioRender. Amodeo, G. (2025) https://BioRender.com/4ak2djy (accessed on 15 October 2025).

Some critical limitations remain. Across several studies, both preclinical and clinical, important details regarding the origin, production, composition and characterization of the secretome were either missing or poorly described, making it difficult to compare results across trials or to establish standardized protocols. This lack of methodological transparency hinders reproducibility and slows the translation of findings into clinical practice. Further efforts are needed to develop standardized protocols for secretome isolation, characterization and storage, as well as a deeper understanding of the active components and mechanisms of action, as well as optimal dosing and administration strategies. Additionally, as of now, no registered clinical trials explicitly investigating the analgesic potential of MSC secretome therapies are available on major platforms such as ClinicalTrials.gov. High-quality, large-scale randomized trials are urgently needed to confirm efficacy. Moreover, regulatory frameworks are still evolving, and further clarification is required to ensure safe, reproducible and clinically applicable use of the MSC secretome. Indeed, MSC secretome therapies still face major regulatory uncertainty. In both the U.S. and EU, no harmonized guidelines exist for the classification, manufacturing or clinical use of secretome-based products. They may, in fact, be considered as biological medicinal products, advanced therapy medicinal products (ATMPs), or they may fall under other frameworks depending on their preparation. Although regulatory frameworks exist for general biological products, agencies such as the U.S. FDA (Food and Drug Administration) and EMA (European Medicines Agency), no product-class-specific guidance has yet been finalized for MSC secretome products. Therefore, the challenges related to batch consistency, GMP production and quality control remain open issues [144,145,146]. In this regard, it should be noted that other cell-free approaches, such as peptide-based therapies and synthetic exosomes, are also under investigation. Unlike MSC-derived products, these strategies allow greater chemical standardization [147]. However, they lack the pleiotropic and multi-target activity as well as the regenerative properties of the biological secretome. Finally, to promote transparency and reproducibility across studies, it is needed that future investigations report a minimal set of methodological and compositional parameters for MSC secretome research (Figure 3). Such a checklist could facilitate data comparison, improve quality control and accelerate translation from preclinical to clinical applications.

In conclusion, MSC secretome therapy represents a rapidly advancing and innovative field in pain medicine. Although preclinical findings strongly support its analgesic potential, clinical evidence is still in its early stages and should be interpreted with caution until validated by robust, large-scale randomized trials. However, with continued research and translational efforts, this cell-free approach holds significant potential to become a safe, effective and widely accessible therapeutic option for managing chronic pain across diverse clinical settings.

## Figures and Tables

**Table 1:** Source and properties of MSCs.

Source	Tissue	Abbreviation	Proliferation	Immunomodulation	Differentiation Potential	Collection Ease	Clinical/Research Status	Notes	Ref.
**Perinatal**	Umbilical cord (Wharton’s Jelly)	UC-MSC/ WJ-MSC	High	Very high	Good	Non-invasive	In clinical trials	Collected at birth	[59]
	Umbilical cord blood	UCB-MSC	Low	High	Limited	Non-invasive	Rarely used	Contains fewer MSC than WJ	[60]
	Amniotic membrane	AM-MSC	High	Very high	Medium	Surgical discard	Interest in ophthalmology, wound healing	inner fetal membrane, non-tumorigenic	[61]
	Amniotic fluid	AF-MSC	High	High	High (pluripotent-like)	Only during pregnancy	Advanced experimental	Isolated in mid- to late pregnancy	[62]
	Placenta (chorion and decidua)	PL-MSC	High	High	Good	Postpartum recovery	Under clinical study	Both fetal and maternal origin	[63]
	Chorionic villi	CV-MSC	Medium	High	High	Invasive	Mostly in research	Rarely used but high potential	[64]
**Adult**	Bone marrow	BM-MSC	Medium	High	High	Invasive	Clinically used	Gold standard MSC	[65]
	Adipose tissue	AD-MSC	High	High	Good	Easy	Clinically used	Abundant; minimally invasive harvesting (lipoaspirate)	[66]
	Skeletal muscle	SM-MSC	Medium	Medium	Osteo-myogenic	Invasive	Experimental	Rarely used; complex isolation	[67]
	Periosteum/cortical bone	PO-MSC	Medium	Medium	High (bone)	Invasive	Preclinical	Rich in osteoprogenitors; bone regeneration	[68]
	Synovial membrane	SF-MSC (adult)	Medium	High	High chondrogenic	Invasive	Preclinical/early translational research (OA)	Harvested via biopsy or arthroscopy	[69]
	Synovial fluid	SF-MSC (early life)	Medium-High	Medium-High	Good (incl. chondrogenic)	Minimally invasive; rare sample	Very limited; early preclinical only	Low in quantity; harvested during joint surgeries	[70]
	Articular cartilage	N/A	Low	N/A	High chondrogenic	Not easily accessible	Highly experimental	Difficult to isolate	[71]
	Meniscus	N/A	Low	Medium	Fibrocartilaginous	Surgical	Early-stage research	Specific to meniscal repair	[72]
	Liver	Hepatic MSC	Low	High	Hepatogenic	Invasive	Preclinical	Harvested via biopsy	[73]
	Lung	Pulmonary MSC	Medium	High	Pulmonary	Invasive	In respiratory research models	Present in alveolar tissue, harvested via biopsy	[74]
	Dental pulp (including the one of deciduous tooth)	DP-MSC (SHED)	High	High	Good	Minimally invasive (Easy, natural shedding)	Under investigation, in clinical development	From either deciduous or permanent teeth	[75]
	Gingival tissue	GMSC	High	High	Good	Easy	Dental studies	Easily accessible	[76]
	Salivary and parotid glands	SG-MSC	Medium	High	Good	Surgical biopsy	Experimental	High plasticity	[77]
	Endometrium	EM-MSC	High	High	Vascular, mesodermal	Cyclical collection	Under research	Cyclically regenerating MSCs	[78]
	Peripheral blood	PB-MSC	Very low	Medium	Limited	Non-invasive	Low yield	Extremely rare	[79]
**Alternative**	Urine	USCs	High	High	Urologic, muscular	Non-invasive	In development	Promising for urological and nephrological applications	[80]
	Breast milk	N/A	High	High	Good	Non-invasive	Very experimental	Multilineage potential; currently under investigation	[48]
	Ovarian/Testicular tissue	N/A	Medium-High	High	Gonadal support (follicle, sperm development)	Invasive biopsy	Preclinical (ovarian regeneration)	Very preliminary studies	[81]
	Inflamed synovial fluid (OA, RA)	N/A	Medium–Low (senescence in RA)	Medium–High (inflammation-enhanced)	High (chondrogenic)	Arthrocentesis, low and variable yield	Research (OA/RA)	Potential therapeutic auto-feedback mechanism	[82]
	Menstrual blood	MB-MSCs	High	High	Good	Very easy	Promising	High proliferation rate	[83]

MSC(s): mesenchymal stem cell(s); WJ: Wharton’s jelly; OA: osteoarthritis; RA: rheumatoid arthritis; N/A: not available.

**Table 2:** Effects of secretome therapy in rodent pain models.

Pain Model	Secretome Source	Type	Processing and Storage	Treatment Time After Model Induction	Dose	Route and Volume	Effect on Pain	Biochemical Evaluations: Time and Effect	Ref.
**STZ (mouse)**	human ASC	whole	Processing: concentrated Storage: −80 °C	W2	2 × 10^6^ (single dose)	Intravenous, 200 µL	↓ Mechanical allodynia (von Frey test)	W3 and W14—PNS and CNS: ↓ Pro-inflammatory cytokines ↑ Anti-inflammatory cytokines	[102]
				W6	2 × 10^6^ (single dose)				
				W2 + W6	2 × 10^6^ (total dose 4 × 10^6^)			N/A	
**CIPN-oxaliplatin (rat)**	rat ASC	whole	Processing: freeze-dried Storage: frozen	W2	1 × 10^6^ (single dose)	Intraperitoneal, 3.5 mL	¬ Weight-bearing changes (incapacitance test)	N/A	[103]
**STZ (mouse)**	mouse BM-MSC	whole	Processing: concentrated Storage: −80 °C	W4	1 × 10^6^ (single dose)	Intravenous, 100 µL	↓ Mechanical allodynia (von Frey filaments) ↓ Thermal hypoalgesia (Plantar test)	N/A	[104]
**PNL (mouse)**	mouse BM-MSC	whole	Processing: concentrated Storage: −80 °C	W1	1 × 10^6^ (single dose)	Intravenous, 100 µL	↓ Mechanical allodynia (von Frey filaments) ↓ Thermal hyperalgesia (Plantar test)	W3—PNS and CNS: ↓ Pro-inflammatory cytokines ↑ Anti-inflammatory cytokines	[105]
**K-OA—collagenase type VII (mouse)**	human BM-MSC	whole	Processing: concentrated Storage: −80 °C	W1 (3 treatments: one every two days)	2 × 10^4^ (total dose 6 × 10^4^)	Intra-articular, 6 µL	↓ Weight-bearing changes (incapacitance test)	W4—OA knee: ¬ modulation on subchondral bone volume, synovial membrane thickness and synovial inflammation	[106]
**SNL (rat)**	human UC-MSC	EVs	Processing: concentrated Storage: −80 °C	W0 (day 3 post SNL)	0.12 mg/mL (single dose)	Intrathecal catheter, 10 µL	Acute effect: ↓ Dose–response of mechanical allodynia (von Frey filaments) ↓ Dose–response of thermal hyperalgesia (hot plate test)	N/A	[107]
					0.6 mg/mL (single dose)				
					1.2 mg/mL (single dose)				
				W0 (day 8 post SNL)	0.12 mg/mL (single dose)	Intrathecal catheter, 10 µL	Acute effect: ↓ Dose–response of mechanical allodynia (von Frey filaments) ↓ Dose–response of thermal hyperalgesia (hot plate test)	N/A	
					0.6 mg/mL (single dose)				
					1.2 mg/mL (single dose)				
				W0 (preventive treatment) (one treatment every day for 8 days from the SNL day)	1.2 mg/mL (total dose 9.6mg/mL)	Intrathecal catheter, 10 µL	Prevents the onset of: Mechanical allodynia (von Frey filaments) Thermal hyperalgesia (hot plate test)	W1—PNS and/or CNS: ↓ Pro-inflammatory cytokines ↑ Anti-inflammatory cytokines ↓ Glia activation	
				W0 (therapeutic treatment) (one treatment every day for 8 days from day 4 after SNL)	1.2 mg/mL (total dose 9.6 mg/mL)	Intrathecal catheter, 10 µL	↓ Mechanical allodynia (von Frey filaments) ↓ Thermal hyperalgesia (hot plate test)	N/A	
**db/db mice**	human ASC	whole	Processing: concentrated Storage: N/A	W18 + W20 + W22 + W24	1 × 10^9^ (total dose 4 × 10^9^)	Intravenous, 50 µL	↓ Mechanical allodynia (von Frey test) ↓ Thermal hyperalgesia (plantar test)	W26—PNS: ↑ Intraepidermal nerve fiber density ↓ Pro-inflammatory markers, neurodegeneration and apoptosis	[108]
**db/db mice**	mouse BM-MSC	EVs	Processing: concentrated Storage: −80 °C or freshly used	W20 + W21 + W22 + W23 + W24 + W25 + W26 + W27 (8 treatments: one a week) (see Note 1)	1 × 10^9^ particles (total dose: 8 × 10^9^ particles)	Intravenous, N/A	↓ Mechanical allodynia (von Frey filaments) ↓ Thermal hyperalgesia (plantar test)	W28—PNS: ↓ Pro-inflammatory cytokines, ↓ Neurovascular dysfunction and axonal demyelination, ↑ neurological outcomes	[109]
**K-OA—MIA (mouse)**	human ASC	whole	Processing: concentrated Storage: −80 °C	W1	2 × 10^6^ (single dose)	Intravenous, 200 µL	↓ Mechanical allodynia (von Frey test) ↓ Thermal hyperalgesia (Plantar test). Note: IV has a greater pain-relieving effect, followed by IPL and IA	W3—PNS and CNS: ↓ Pro-inflammatory cytokines, macrophages/microglia markers, GFAP and ATF3. *Note: IV has a greater pain-relieving effect, * *followed by IPL and IA*	[110]
						intrarticular, 15 µL			
						Intraplantar, 15 µL			
**db/db mice**	mouse BM-MSC	EVs	Processing: concentrated Storage: −80 °C or freshly used	W20 + W21 + W22 + W23	1 × 10^9^ particles (total dose 4 × 10^9^ particles)	Intravenous, N/A	↓ Mechanical allodynia (von Frey filaments) ↓ Thermal hyperalgesia (plantar test)	W24—PNS: ↓ inflammatory macrophages markers ↑ neurological function and recovery	[111]
**CCI (rat)**	rat BM-MSC	whole	Processing: N/A Storage: −80 °C	W0 (preventive treatment—one day before CCI and after 7 and 11 days)	N/A	Intraperitoneal, 1 mL	↓ Mechanical allodynia (von Frey filaments) ↓ Thermal hyperalgesia (hot plate test)	W2—CNS: ↓ Purinergic receptors (P2X4 and P2X7)	[112]
**SNL (rat)**	Thera101 (purchased from Theratome Bio, Inc.)	N/A	N/A	W0 (preventive treatment)	1–2 mg/kg	Directly to the injured nerve, 350–450 µL	↓ Mechanical allodynia (von Frey test) *only acute evaluations (30, 60, 120 min)*	N/A	[113]
**PNL (mouse)**	human SHED	whole	N/A	W0: every day for a week	1 × 10^5^ (total dose 7 × 10^5^)	Intravenous, N/A	↓ Mechanical allodynia (von Frey test)	W1—PNS and CNS: ↓ Pro-inflammatory cytokines and microglia markers ↑ Anti-inflammatory markers	[114]
				W1: every day for a week	1 × 10^5^ (total dose 7 × 10^5^)	Intravenous, N/A	↓ Mechanical allodynia (von Frey test)	N/A	
				W2: every day for a week	1 × 10^5^ (total dose 7 × 10^5^)	Intravenous, N/A	↓ Mechanical allodynia (von Frey test)	N/A	
**similar to CCI damage induced by biomaterial implant containing TNF (rat)**	rat ASC	whole	Processing: concentrated Storage: N/A	W0: biomaterial implant containing secretome in combination with TNF (preventive treatment)	N/A	Biomaterial implant at sciatic nerve level	N/A	W3—PNS: prevention of nerve demyelination and morphological alteration	[115]
**IC(rat)**	human UC-MSC	EVs	N/A	W1: 3 doses on alternate days	20 μg (total dose 60 μg)	Intrathecal, 20 µL	↓ Mechanical allodynia (von Frey filaments)	N/A—CNS: ↓ Pro-inflammatory cytokines and glia markers	[116]
**TMJ-OA—MIA (rat)**	human DPSC	whole	Processing: concentrated Storage: −80 °C	W4 + W5 + W6 (i.e., once a week for 3 weeks)	N/A	N/A, 100 µL	↓ Mechanical allodynia (von Frey filaments)	W8 and W12—TMJ: ↓ inflammation ↑ extracellular matrix and subchondral bone repair and regeneration	[117]
**CCI (rat)**	human UC-MSC	EVs	Processing: concentrated Storage: −80 °C	W0: on day 2, 4 and 6 after CCI	5 µg (total dose 15 µg)	Intrathecal, 25 µL	↓ Mechanical allodynia (von Frey filaments)	W1: ↓ Pro-inflammatory cytokines and microglia markers	[118,119]
**CCI (rat)**	rat BM-MSC	whole	Processing: N/A Storage: none, freshly used	W0 (preventive treatment—for 3 consecutive days from the day pre-CCI)	N/A	Intraperitoneal, 1 mL	↓ Mechanical allodynia (von Frey filaments) ↓ Thermal hyperalgesia (hot plate test)	W2: CNS ↓ Pro-inflammatory cytokines	[120]

STZ: streptozotocin-induced diabetic neuropathy model; CIPN: chemotherapy-induced peripheral neuropathy model; PNL: partial nerve ligation-induced neuropathy model; SNL: spinal nerve ligation-induced neuropathy model; CCI: chronic constriction injury-induced neuropathy model; MIA: monoiodoacetate; OA: osteoarthritis; K-OA: knee osteoarthritis model; TMJ-OA: temporomandibular joint osteoarthritis model; IC: interstitial cystitis bladder pain syndrome model; ASC: adipose-derived mesenchymal stem cells; BM-MSC: bone marrow-derived mesenchymal stem cells; UC-MSC: umbilical cord-derived mesenchymal stem cells; SHED: stem cells from human exfoliated deciduous teeth; DPSC: dental pulp stem cells; EVs: extracellular vesicles–exosomes; W: week; PNS: peripheral nervous system; CNS: central nervous system; TNF: tumor necrosis factor; GFAP: glial fibrillary acidic protein; ATF3: activating transcription factor 3; IV: intravenous; IA: intrarticular; IPL: intraplantar; ↑: increase; ↓: decrease; ¬: no changes; N/A: not available. Note 1: Since this is a spontaneously developing model, in this case, the “W” indicates the age of the animal and not the time after the onset of the disease.

**Table 3:** Effects of secretome therapy on in vitro pain models.

Model	Secretome Source	Type and Administration Method	Effect	Ref.
**DRG neurons from naive rats exposed to high glucose concentrations** **(diabetic polyneuropathy)**	human ASC	whole, in vitro	↓ apoptosis	[130]
**Ex vivo** **: DRG neurons isolated from db/db mice** **(diabetic polyneuropathy)**	human ASC	whole, in vivo	↑ neurite arborization	[108]
**DRG neurons isolated from db/db mice** **(diabetic polyneuropathy)**	human ASC	whole, in vitro	↑ neurite arborization	[131]
**Cerebral organoids (CeO)**	human BM-MSC	whole, in vitro	↑ MOR expression ↑ neurogenesis and astrogenesis	[132]
**Human AF cells from IS or DD patients, stimulated with mechanical stress, IL-1** **β** **or combined** **(chronic back pain)**	N/A	whole, in vitro	↓ inflammation ↑ tissue regeneration	[133]
**AF and NP cells from healthy donors stimulated with TNF-** **α** **(chronic back pain)**	human ASC	whole, EVs or EV-free secretome, in vitro	↓ degeneration	[134]
**Immortalized microglial cell line stimulated with LPS** **(generic model of neuroinflammation/pain)**	human UC-MSC	whole, in vitro	↓ pro-inflammatory cytokines and microgliosis	[118]

DRG: dorsal root ganglia; AF: annulus fibrosus cells; IS: idiopathic scoliosis; DD: disc degeneration; IL-1β: interleukin 1 beta; NP: nucleus pulposus cells; TNF-α: tumor necrosis factor alpha; LPS: lipopolysaccharide; ASC: adipose-derived mesenchymal stem cells; BM-MSC: bone marrow–derived mesenchymal stem cells; UC-MSC: umbilical cord-derived mesenchymal stem cells; EVs: extracellular vesicles–exosomes; MOR: mu opioid receptor; N/A: not available; ↑: increase; ↓: decrease.

**Table 4:** Effects of secretome treatment in patients.

Type of Study	Disease	Secretome Source	Secretome Origin	Treatment Strategy	Treatment Effect	Ref.
N/A	MSK pain	ASC	Self-produced	Local administration. First treatment: 27 body areas; second treatment: 7 sites.	Pain assessed with NRS: pain reduction after 15 min, which was maintained up to 4 weeks later.	[140]
Open-label clinical study	OA knee	UC-MSC	Purchased, GMP certified.	Intra-articular administration (once weekly for 5 weeks).	Reduction of VAS and WOMAC scores up to 12 weeks post-treatment. Reduction of serum MMP-3 levels and an increase in TGF-β levels.	[141]
Case report	CLBP	UC-MSC	N/A	Unique multi-site treatment (in vertebrae from T12 to S5, epidural region of the sacral spine and piriformis muscles).	Before administration, the patient underwent PLDD. Pain reduction lasted for up to six months. The impact of the secretome alone cannot be determined in a control group (no treatment).	[142]
Randomized controlled clinical trial	TBPI	UC-MSC	Self-produced	Single administration at the neuromuscular junction of the median nerve-superficial flexor digitorum muscle.	Before administration, patients underwent nerve transfer surgery. A reduction in postoperative pain was documented. The impact of the secretome cannot be determined due to the lack of a control group (no treatment).	[143]

MSK: musculoskeletal; OA: osteoarthritis; CLBP: chronic low back pain; TBPI: total brachial plexus injury; ASC: adipose-derived mesenchymal stem cell; UC-MSC: umbilical cord-derived mesenchymal stem cell; GMP: good manufacturing practice; T: thoracic; S: sacral; NRS: numeric rating scale; VAS: visual analog scale; WOMAC: Western Ontario and McMaster universities arthritis index; MMP-3: matrix metalloproteinase-3; TGF-β: transforming growth factor-beta; PLDD: percutaneous laser disc decompression; N/A: not available.

**Table 5:** Clinical trials assessing the effects of secretome on pain.

NCT Number	Condition	Secretome Source	Secretome Origin	Treatment Strategy	Pain Measurement	Study Status
NCT05909488	RP	UC-MSC	N/A	Peribulbar injection of 1.5 or 5 × 10^6^ UC-MSCs resuspended in CM	6 months after injection: evaluation of the level of pain felt by patients	not yet recruiting
NCT06688318	OA knee	UC-MSC	N/A	Intra-articular injection of CM from hypoxic UC-MSCs	2, 4 and 6 months after injection: KOOS and WOMAC scores	active, not recruiting
NCT05579665	OA knee	UC-MSC	N/A	Intra-articular injection under ultrasound guidance of CM from UC-MSCs (once weekly for 5 weeks)	pre-treatment, and 3 and 6 months after injection: VAS and WOMAC scores	completed [141]
NCT04314661	OA knee	UC-MSC	N/A	Intra-articular injection of 10 × 10^6^ UC-MSCs and 2 cc secretome twice with a 2-week interval, or 2 cc secretome, 10 × 10^6^ UC-MSCs and 2 cc secretome with a 2-week interval	1, 3 and 6 months after injection: VAS and WOMAC scores	unknown status

RP: retinitis pigmentosa; OA: osteoarthritis; UC-MSC(s): umbilical cord-derived mesenchymal stem cell(s); N/A: not available; CM: conditioned medium; cc: cubic centimeter, i.e., mL; KOOS: knee injury and osteoarthritis outcome score; WOMAC: Western Ontario and McMaster universities arthritis index; VAS: visual analog scale.

## Data Availability

No new data were created or analyzed in this study. Data sharing is not applicable to this article.

## Supplementary Materials

The following supporting information can be downloaded at: https://www.mdpi.com/article/10.3390/brainsci15121263/s1. Table S1: list of clinical trials retrieved from ClinicalTrials.gov on 28 August 2025, using the keywords secretome and conditioned medium with the study type filter set to interventional. The search yielded 87 trials, of which 52 remained after manual review. The four trials evaluating pain outcomes are highlighted in red.

## Abbreviations

The following abbreviations are used in this manuscript:

AF	Annulus Fibrosus
AKT	Protein Kinase B
ASCs	Adipose-derived Stromal/Stem Cells
ATMPs	Advanced Therapy Medicinal Products
BDNF	Brain-Derived Neurotrophic Factor
CD55	Cluster of Differentiation 55
CeOs	Cerebral Organoids
CIPN	Chemotherapy-Induced Peripheral Neuropathy
CM	Conditioned Medium
DASH	Disabilities of the Arm, Shoulder and Hand
DOR	Delta (δ) Opioid Receptor
KOR	Kappa (κ) Opioid Receptor
DRG	Dorsal Root Ganglion
EMA	European Medicines Agency
ESCs	Embryonic Stem Cells
EU	European Union
EVs	Extracellular Vesicles
FDA	Food and Drug Administration
FGF2	Fibroblast Growth Factor 2
GDNF	Glial Cell Line-Derived Neurotrophic Factor
GMP	Good Manufacturing Practice
HA	Hyaluronic Acid
IGF	Insulin-like Growth Factor
IL-10	Interleukin 10
IL-1β	Interleukin 1 beta
IL-6	Interleukin 6
IL-8	Interleukin 8
iPSCs	Induced Pluripotent Stem Cells
KL	Kellgren and Lawrence Scale
L2-L4	Lumbar Vertebrae L2 to L4
LPS	Lipopolysaccharide
miRNAs	microRNAs
MMP1	Matrix Metalloproteinase-1
MMP3	Matrix Metalloproteinase-3
MOR	Mu (µ) Opioid Receptor
MSCs	Mesenchymal Stem/Stromal Cells
mTOR	Mammalian Target of Rapamycin
MyD88	Myeloid Differentiation Primary Response 88
Nav1.7 or Nav1.8	Voltage-gated Sodium Channel subtypes 1.7 and 1.8
NF-κB	Nuclear Factor kappa B
NGF	Nerve Growth Factor
NLRP3	NOD-like receptor family pyrin domain-containing 3
NOD	Nucleotide-binding Oligomerization Domain
NRS	Numeric Rating Scale
NSAIDs	Non-Steroidal Anti-Inflammatory Drugs
NSCs	Neural Stem Cells
OA	Osteoarthritis
P2X4/P2X7	Purinergic Receptor P2X4 and P2X7
PBMCs	Peripheral Blood Mononuclear Cells
PET	Positron Emission Tomography
PGE_2_	Prostaglandin E2
PI3K	Phosphoinositide 3-Kinase
PLDD	Percutaneous Laser Disc Decompression
RNA	Ribonucleic Acid
Rsad2	Radical S-Adenosyl Methionine Domain-containing 2
SNRIs	Serotonin-Norepinephrine Reuptake Inhibitors
STZ	Streptozotocin
tDCS	Transcranial Direct Current Stimulation
TGF-β	Transforming Growth Factor beta
TIMP1	Tissue Inhibitor of Metalloproteinases 1
TIMP2	Tissue Inhibitor of Metalloproteinases 2
TLR2	Toll-like Receptor 2
TLRs	Toll-like Receptors
TMS	Transcranial Magnetic Stimulation
TNF-α	Tumor Necrosis Factor alpha
TRPV1	Transient Receptor Potential Vanilloid 1
U.S.	United States
UC-MSC	Umbilical Cord-derived Mesenchymal Stem Cells
VAS	Visual Analog Scale
VEGF	Vascular Endothelial Growth Factor
Wnt	Wnt signaling pathway
WOMAC	Western Ontario and McMaster Universities Osteoarthritis Index
